# Inhibitory effects of total triterpenoids isolated from the *Hedyotis diffusa* willd on H1975 cells

**DOI:** 10.3389/fphar.2022.922477

**Published:** 2022-09-14

**Authors:** Kenan Wu, Xue Wu, Yanni Liang, Ting Wang, Dongzhi Wu, Luhan Li, Zheng Wang

**Affiliations:** ^1^ Shaanxi Collaborative Innovation Center of Chinese Medicinal Resources Industrialization, State Key Laboratory of Research and Development of Characteristic Qin Medicine Resources (Cultivation), Shaanxi University of Chinese Medicine, Xian Yang, China; ^2^ Medical Experiment Center, Shaanxi University of Chinese Medicine, Xian Yang, China

**Keywords:** *Hedyotis diffusa* willd., total triterpenes, H1975 cell, apoptosis, proliferation, NF-κB, STAT3

## Abstract

It is estimated that non-small-cell lung cancer (NSCLC) is responsible for 80% of human deaths related to lung cancer worldwide. Currently, it has been discovered that two transcription factors. Nuclear factor-κB (NF-κB) and Signal transducer and activator of transcription 3 (STAT3) were the main factors affecting inflammation and cancer, and their activation promoted lung cancer cell proliferation. *Hedyotis diffusa* Willd. (*H. diffusa*) is an herbal Chinese medicine, which has always been used for the treatment of malignant tumors in clinical. Previous research found that *H. diffusa* could inhibit the proliferation of H1975 cells, but the specific mechanisms remain elusive. We investigated the effects of total triterpenes extracted from *H. diffusa* (TTH) on the migration, proliferation and apoptosis of H1975 cells. Cell-cycle and immunofluorescence analysis showed that TTH could block H1975 cells at G0/G1 phase and induce apoptosis of experimental cells. The protein levels of Bcl-2 were decreased, while the levels of pro-apoptotic Bax were increased. In addition, TTH could also inhibit the migration of H1975 cells through downregulated MMP-2 and MMP-9 and upregulated TIMP-2. Further research found that the level of phospho-STAT3 was significantly decreased after administration of TTH. And protein expression level of NF-κB in nucleus was decreased after TTH treatment, while NF-κB in cytoplasm increased. These results suggested that TTH could inhibit the proliferation and migration of H1975 cells, and also could induce cell apoptosis. These effects were closely connected to the activation of NF-κB and the phosphorylation of STAT3.

## Graphical Abstract

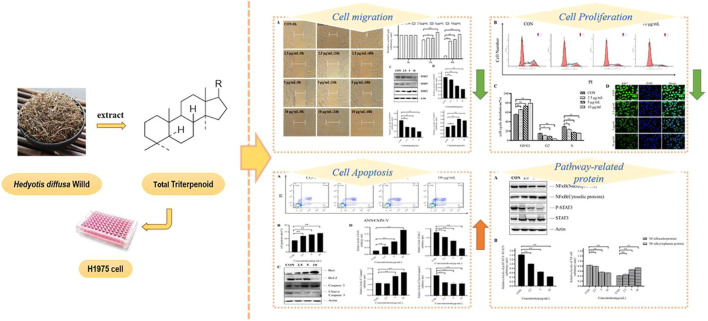

## Introduction

Most cancer deaths result from lung cancer, and its morbidity and mortality remain rank first among malignant tumors (Bray et al., 2018). Non-small cell lung cancer (NSCLC) has the highest incidence of lung cancers, and presents three different histological subtypes, including adenocarcinoma (ADC), squamous cell carcinoma (SCC) and large cell carcinoma (LCC) (Herbst et al., 2018; Suraweera et al., 2020). At present, EGFR and its signal transduction pathway have been studied as therapeutic targets, and a series of reversible EGFR tyrosine kinase inhibitors have been used in the clinical treatment of lung cancer (Choi et al., 2014). However, 20%–30% of lung cancer patients are prone to drug resistance after medication, and the increase of multidrug resistance has a negative impact on the clinical treatment of lung cancer (Wada et al., 2016). Other currently used anticancer drugs usually show unacceptable levels of toxicity to normal cells and tissues, thus limiting the efficacy. At present, there are a lot of literature reports that inflammation plays an important role in the development of many cancers. For instance, the two most classical pathways involved in inflammation of NF-κB and STAT3 could promote lung cancer cell proliferation when activated.

Nuclear factor-κB (NF-κB) is an important nuclear transcription factor in cells. NF-κB regulates tumor proliferation, invasion, angiogenesis and metastasis-related gene expression (El-Nikhely et al., 2020). Many different types of human tumors have misregulated NF-κB, that is, NF-κB activates gene expression to maintain cell proliferation and prevents cells apoptosis. In cancer, the proteins that control NF-κB signal transduction are mutated or abnormally expressed, causing poor coordination between malignant cells and other organisms (Pflug and Sitcheran, 2020). Take into account these evidences, NF-κB is considered to be closely related to the entire process of tumorigenesis. In addition to NF-κB, another pathway associated with inflammation and tumors is STAT3. Evidence shows that Signal Transducers and Activators of Transcription (STAT) family protein STAT3 can regulate the biological behavior of tumor cells and immune cells by mediating the extracellular signals of inflammatory mediators. Moreover, STAT3 is an indispensable key molecule in the process of chronic inflammation promoting tumorigenesis and tumor-related inflammation (Gharibi et al., 2020). In the process of chronic inflammation promoting tumor development, the STAT3 activity of cells can be significantly increased under the stimulation of inflammatory factors such as IL-6, thereby upregulating the expression of cyclins and oncoproteins. And the activation of STAT3 can also upregulate the expression of anti-apoptosis and cell survival-related proteins such as BCL-2, Mcl-1, and survivin, which can significantly promote cell proliferation and reduce cell death (Jing et al., 2020; Lee et al., 2020). It was also reported that activation of STAT3 could promote the expression of MMP2 and MMP9 in tumor cells, thus upregulate their ability of invasion and metastasis (Wang et al., 2021). Studies have shown that many drugs could inhibit NSCLC by regulating NF-κB and STAT3 pathways.

*Hedyotis diffusa* Willd. (*H. diffusa*) is a traditional Chinese medicine, it is distributed in Guangxi, Fujian, Anhui, Yunnan, and other regions of China (Li et al., 2018; Wazir et al., 2021). It was discovered that *H. diffusa* had a variety of pharmacological activities, including anti-bacterial, immunity enhancement, anti-inflammatory, and anti-tumor (Chen et al., 2016). It is widely used in digestive system tumors, reproductive system tumors and other anti-cancer treatments (Wang et al., 2017). It was reported that chemical components in *H. diffusa* mainly included anthraquinones, flavonoids, glycosides, terpenes, volatile components, sterols, polysaccharides, and phenylpropanoids (Niu and Meng, 2013). At present, *H. diffusa* has been used as an adjuvant in the treatment of NSCLC (Lan, 2015). Sun extracted total flavonoids and total anthraquinone from *H. diffusa*, and found that both extracts had anti-tumor activity on lung cancer cell A549 (Sun et al., 2019). Ye found that the water extract of *H. diffusa* has a significant protective effect on kidney tissue, which significantly inhibited the productions of tumor necrosis factor-α (TNF-α), interleukin (IL)-1β, IL-6, as well as obviously promoted the production of IL-10 in serum. The results showed that flavonoids, iridoids and anthraquinones were the active components of the extract of *H. diffusa* on mice kidney tissue (Ye et al., 2015). Some network pharmacological studies have shown that the anti-tumor-related targets of *H. diffusa* are closely related to MAPK-8, STAT3, and MMP9 (Song et al., 2019). Triterpenoids have also been shown to exhibit anti-hepatocellular and colon cancer activity through inhibition of the NF-KB pathway or STAT3 pathway (Verma et al., 2018; Fan et al., 2021). Based on this, we aim to investigate the inhibitory effect of TTH on NSCLC cell line H1975 and whether its potential mechanism is related to NF-κB and STAT3 signal pathway.

## Materials and methods

### Plant materials

Authentic plant material was purchased from Shaanxi Xing Sheng De Pharmaceutical Co., Ltd. (Shaanxi, China, Lot:20181201). Identification of the *H. diffusa* Willd. herb was confirmed by Dr Liu Shijun (Department of Processing, Shaanxi University of Traditional Chinese Medicine, Shaanxi, China).

### Preparation of the extracts

*H. diffusa* Willd. (2.5 Kg) were extracted two times (each for 2 h) with 80% EtOH (100 L) under reflux. After evaporation of the combined ethanol extracts in vacuo, the resultant aqueous residues were dissolved in water and then extracted successively with petroleum ether and ethyl acetate to obtain the petroleum ether extract and the EtOAc extract. Once the solvent was removed, the residue was dissolved in distilled water and placed in the fridge overnight. The next day, the supernatant was collected and then subjected to column chromatography on macroporous resin (HPD-826, 2 BV/h), eluted with 80% aqueous EtOH. 80% ethanol extract was purified again with HPD-826 macroporous resin, eluted with 70% aqueous EtOH. After evaporation of the ethanol extracts in vacuo, ethanol extract was dissolved in anhydrous ethanol, and then activated carbon (10 mg/ml) was added in water for 30°C for decolorization and impurity removal until colorless (each for 30 min). After filtering the activated carbon, the filtrate was added with distilled water to precipitate and centrifuge. Total Triterpenoids were obtained.

### Content determination

Total Triterpenoids content was determined following the procedure described by Wei et al. with slight modifications (Wei et al., 2015). Briefly, after a 600 μl oleanolic acid reference substance and 2 ml sample solution in 10 ml volumetric flask respectively was heated to evaporate in a water-bath at 80°C, 0.2 ml of 5% vanillin-acetic solution and 0.8 ml of perchloric acid were added accurately, and incubated at 70°C water-bath for 15 min. Then the mixed solution was cooled in ice water for 5 min. Then, 5 ml glacial acetic acid was added, shaken well, and stood for 5 min. The absorbance was measured at 548 nm against blank using a spectrophotometer. The content was determined using the oleanolic acid standard calibration curve.

### Cell culture and MTT assay

H1975, A549, and SW620 cell lines were purchased from Fenghui Biotechnology Co., Ltd. (Hunan, China). Cells were cultured in 90% RPMI-1640 (Solarbio, Beijing, China) media supplemented with 10% fetal calf serum (Corning), 100 U/ml penicillin and 100 U/ml streptomycin, and with 5%(v/v) CO_2_ in 37°C incubator for 24 h.

Cells were inoculated with a density of 1.5×10^5^ cells/well into 96-well plates. After 24 h, H1975 (A549, SW620) cells were treated with different concentrations of TTH (1 µg/ml, 2 µg/ml, 5 µg/ml, 10 µg/ml, 15 µg/ml, 20 µg/ml, 30 µg/ml, 50 µg/ml and 75 µg/ml) for 24 and 48 h, respectively. Then the cells were cultured with 20 μl MTT solution for 4 h (5 mg/ml). The MTT solution was removed and 150 µl of DMSO. After thorough mixing, absorbance of each well with a microplate reader (A490) (Molecular Device, Sunnyvale, CA, United States). Half-maximal inhibitory concentration (IC_50_) values were calculated by SPSS.

### *In vitro* scratch assay

When at 100% confluence, the cells followed by starvation in serum-free RPMI 1640 medium for 12 h to completely inhibit cell proliferation. A 200-μl sterile pipette tip was used to make a line in cell cultures. Cells were then incubated with different concentrations of TTH for 0, 24, and 48 h in incubators. The scratch gap width in each group was measured at different positions and compared to the gap width at 0 h which was arbitrarily set at 1.

### Immunofluorescence in H1975 cells

H1975 cells culture on glass cover slips placed in 6-well dishes. After treatment cells were washed with PBS twice, fixed with 4% paraformaldehyde for 10 min, and permeabilized with 1% Triton X-100 for 10 min. After being sealed with 5% BCA for 40 min, cells were incubated with anti-Ki67 (1:100) primary antibody at 4°C overnight. The next day, cells were incubated with a secondary antibody conjugated with FITC-conjugated secondary antibody (Invitrogen) for 1 h, and then mounted by using Prolong Gold Anti-fade reagent with DAPI (Boster) for 5 min. Immunofluorescence micrographs were produced by using an Olympus FSX100 Fluorescence Microscope (Olympus, Tokyo, Japan).

### Western blot analysis

H1975 cells were washed with ice-cold PBS and lysed using RIPA buffer supplemented with protease and phosphatase inhibitor mixtures (Boster Biological Technology Co., Ltd., Wuhan, China) on ice. The Lysates were homogenized twice in 1.5 ml EP tube, 5–10 s each time, and then lysed on ice for 20 min. Lysates were separated by centrifugation at 4°C and 10,000 g for 10 min. Protein concentration was determined by BCA assay (Beyotime Biological Technology Co., Ltd., Shanghai, China). Finally, add loading buffer in proportion to the protein and store at −20°C. 40 mg total proteins were subjected to sodium dodecyl sulfate-polyacrylamide gel electrophoresis (SDS-PAGE) and transferred to PVDF membranes (Millipore, Bedford, MA, United States). The membranes were blocked for 2 h with 5% non-fat dry milk at room temperature, then incubated with rabbit mAbs specific for TIMP2 (1:500) (Abcam, ab180630), MMP9 (1:1,000) (Proteintech, 00083130), MMP2 (1:1,000) (Proteintech, 00082362), NF-ΚB/p65 (1:1,000) (CST, #8242), *p*-STAT3 (1:1,000) (CST, #9145), STAT3 (1:1,000) (CST, #30835), Bax (1:1,000) (Abcam, ab69643), Bcl-2 (1:1,000) (Abcam, ab32124), anti-caspase3 (1:1,000) (Abcam, ab2171) and anti-cleaved caspase-3 (1:1,000) (Abcam, ab32024) overnight at 4°C. Following washing and subsequent incubation with horseradish peroxidase conjugated secondary antibodies (1:3,000) 37°C for 1 h. Then immunoreactive proteins were visualized using ECL Western blotting detection reagent (Millipore, Billerica, MA, United States) and detected using MultiImage Light Cabinet Filter Positions (Alpha Innotech, San Leandro, CA, United States). ImageJ software version 1.0 was used for gray-scale value analysis and quantification of Western blots.

### Flow cytometry analysis

Cell cycle distribution was analyzed by flow cytometry (Becton, Dickinson and company, Franklin Lakes, NJ, United States). H1975 cells were seeded in a 6-well plate at a density of 1 × 10^6^ cells/well, and divided into control, 2.5 µg/ml, 5 µg/ml and 10 µg/ml groups. After 48 h of treatment, cells were harvested, rinsed with PBS, fixed with 75% (v/v) ice-cold ethanol and re-suspended in staining buffercontaining FITC-Annexin V and propidium iodide (PI). The mixture was then incubated in the dark at 37°C for 30 min. DNA contents of stained nuclei were analyzed, and the cell numbers in each cycle phase were calculated.

Cell apoptosis was detected using Annexin V-FITC Apoptosis Detection Kit I (BD Biosciences, San Diego, CA, United States) according to the manufacturer’s instruction. H1975 cells were treated with different concentrations of TTH for 48 h according to the cell cycle distribution experiment. Cells were washed twice with pre-cooled PBS, then digested with 0.25% trypsin free of EDTA, re-suspended in binding buffer and then incubated with Annexin V-FITC and PI for 10 min at 25°C in dark. Samples were then analyzed by FACS Calibur (BD Bioscience). The percentage of stained cells was determined using BD FACSDiva software (Becton, Dickinson and company, Franklin Lakes, NJ, United States).

### Statistical analysis

Each experiment was repeated at least three times, and the data are expressed as the mean ± SEM. Statistical differences between groups were analyzed by Student’s t-test or the Mann-Whitney *U* test as appropriate using a SPSS 26.0 program. *p < 0.05 was considered statistically significant.

## Results

### Content determination results of the test sample

The standard curve equation was *Y* = 12.27*X*–0.0649 and expressed good linearity (*r* = 1) within the test ranges. According to the results, the content of TTH in 70% EtOH eluates was 83.12 ± 0.85%.

### TTH inhibited H1975 cell proliferation

**FIGURE 1:**
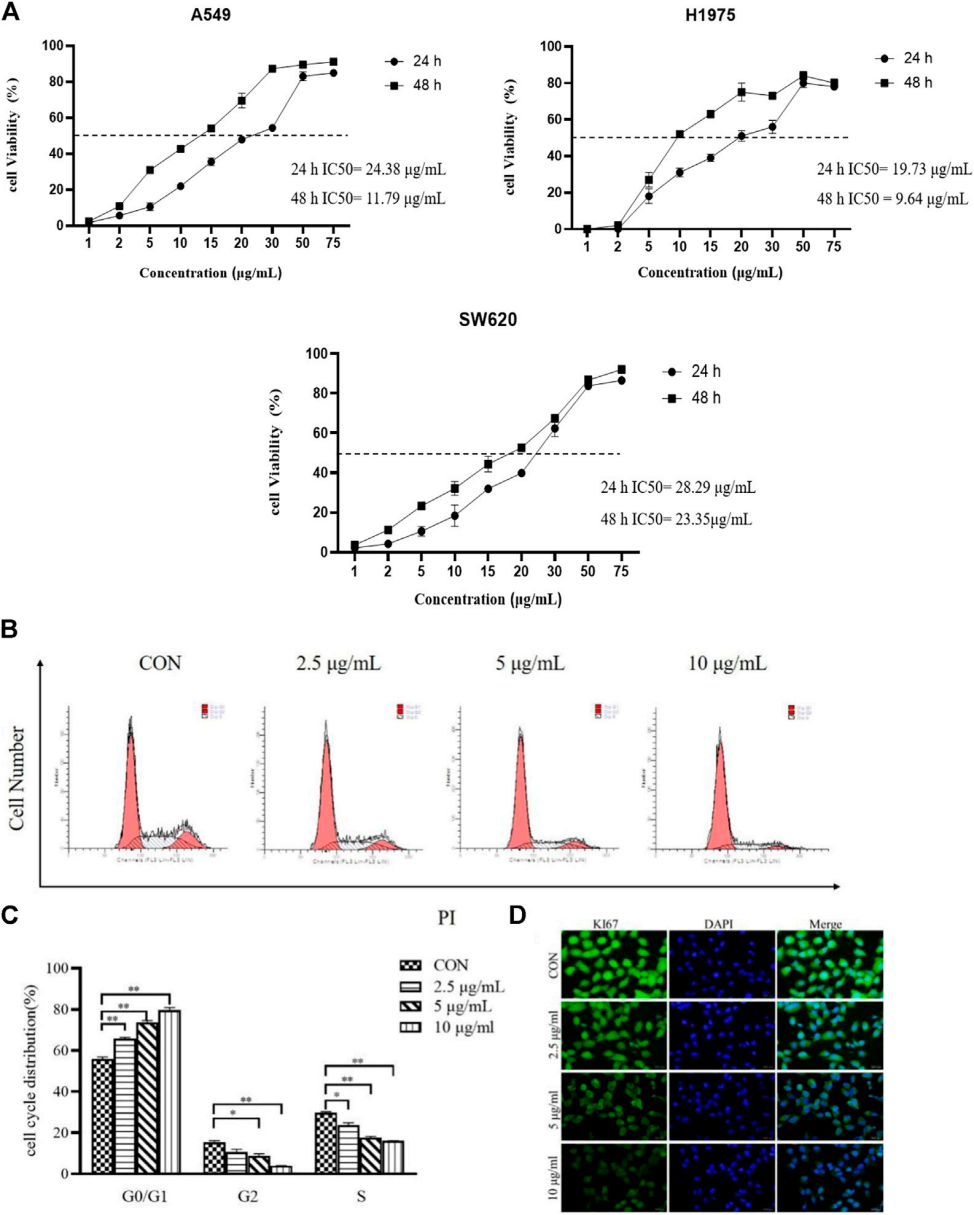
Effects of TTH on proliferation of H1975. **(A)** H1975 (A549,SW620) cells were treated with various concentrations (1, 2, 5, 10, 15, 20, 30, 50, 75 µg/ml) of TTH for 24 and 48 h, respectively. **(B)** H1975 cells were treated with TTH at 2.5 µg/ml, 5 µg/ml and 10 µg/ml for 48 h and the DNA content was analyzed by flow cytometry; **(C)** Histogram summarized the results of **(B)**. **(D)** Immunofluorescence staining of Ki67 on H1975 cell was used to detect the cell proliferative ability after administration. Scale bar = 500 μm. Cell numbers at G0/G1, G2and S phases were counted and the percentage was calculated. Error bars represent means ± SEM of *n* = 3. *p < 0.05 and **p < 0.01 vs. control group.

Cellular-based screening of TTH were performed on A549, SW620, and H1975 cell lines to determine the activity of the derivatives by the MTT assay *in vitro*. The IC50 values obtained are tabulated in Figure 1A. As shown in Figure 1A, after treatment with various concentrations (1, 2, 5, 10, 15, 20, 30, 50, 75 µg/ml) of TTH for 24 and 48 h, the proliferation of H1975 cells were inhibited moderately. Compounds TTH showed significant anticancer activity against A549, SW620, and H1975 with 24 h IC_50_ values of 24.38, 28.29, and 19.73 µg/ml, respectively. The results showed that the IC_50_ value of 48 h was 11.79, 23.35, and 9.64 µg/ml, respectively. In view of the good activity of TTH on H1975 cell lines, the antitumor activity of H1975 was further evaluated.

Cell proliferation plays an important role in tumors, and flow cytometry was used to analyze cell cycle distribution. As shown in Figures 1B,C, TTH significantly increased the number of cells in the G0/G1phase. Contrarily, the number of cells in the S phase were decreased along with the increase of the treatment concentration. The results showed that TTH could significantly arrest cell cycle at G0/G1 phase.

As showed in Figure 1D, the expression of Ki-67 decreased in H1975 cells *in vitro* after administration compared with control cells. The results showed that total Triterpenoids inhibited the proliferation of H1975 cells.

### TTH induced apoptosis and regulateds apoptosis-related proteins in H1975 cells

**FIGURE 2:**
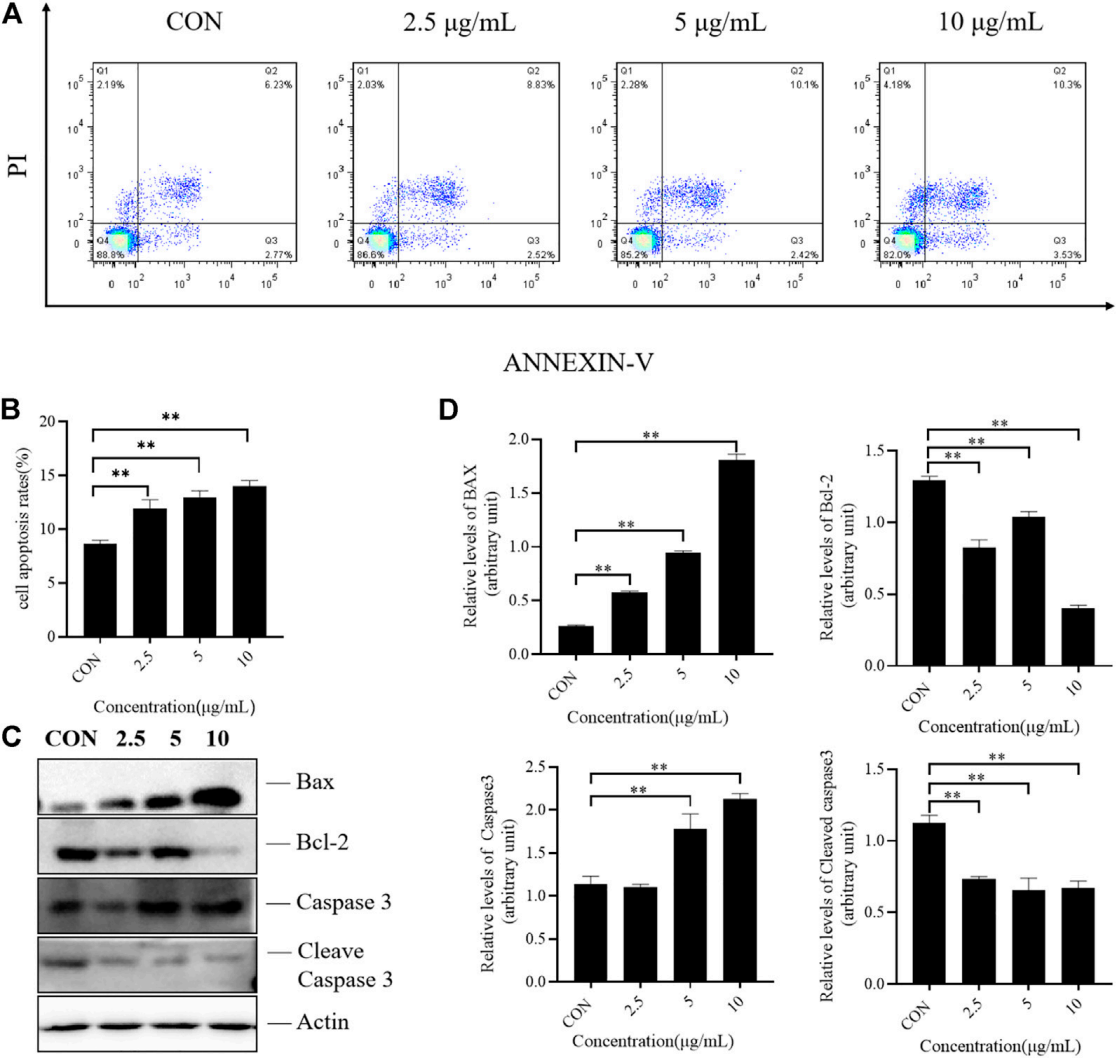
Effects of TTH on the apoptosis of H1975. **(A)** H1975 cells were treated with TTH at 2.5 µg/ml, 5 µg/mL and 10 µg/ml for 48 h and cell apoptosis was analyzed by flow cytometry; **(B)** Histogram summarized the results of **(A)**. The activity of H1975 was evaluated by Annexin-V/PI staining; **(C)** Western blot showed the protein level of caspase-3, cleaved caspase-3, Bax and Bcl-2 following the total Triterpenoids treatment for 48 h. Actin served as an equal loading control; **(D)** Histogram summarized the results of **(C)**. Error bars represent means ± SEM of *n* = 3. **p < 0.01 vs. control group.

Flow cytometry was used to detect the apoptosis of H1975 cells treated with different doses of TTH for 48 h. As shown in Figure 2A, Annexin V-negative/PI negative cells (Q4 quadrant) represented viable cells; Annexin V-positive/PI positive cells (Q2 quadrant) indicated cells undergoing late-stage apoptosis; and Annexin V-positive/PI negative cells (Q3 quadrant) were considered to represent early apoptosis cells. Q2 and Q3 represented the apoptosis cells. After treatment with TTH for 48 h, cells in Q2 and Q3 were greatly increased, indicating that TTH could induce the apoptosis of H1975 cells.

Apoptosis is a complex and multi-stage process involving multiple genes. Due to the different initial stages of apoptosis, there are three pathways. The mitochondrial pathway is the first stronghold of apoptosis. This pathway includes the interaction between the Bcl-2 family containing the BH3 domain and the Bcl-2 family bound to the outer mitochondrial membrane, which changes the permeability of mitochondrial membrane, activates caspase pathway and induces apoptosis. In the present study, the protein expression levels of caspase-3, cleaved caspase-3, Bax and Bcl-2 were determined by Western blotting. As shown in Figures 2C,D, the results revealed the expression of Bcl-2 were decreased, while that of proapoptotic protein Bax were increased after TTH treatment. These results indicated that TTH could regulate the expression of Bcl-2/Bax and induce apoptosis. However, it had little effect on the activation of Caspase3.

### TTH inhibiteds migration of H1975 cells

Cell migration plays a central role in many physiological and pathological processes such as embryonic development, immune defense, injury repair, angiogenesis, and tumor metastasis. It exists in normal physiological processes such as tissue development, wound healing and rebirth. And it is also a key link in pathological development such as inflammation and tumor.

**FIGURE 3:**
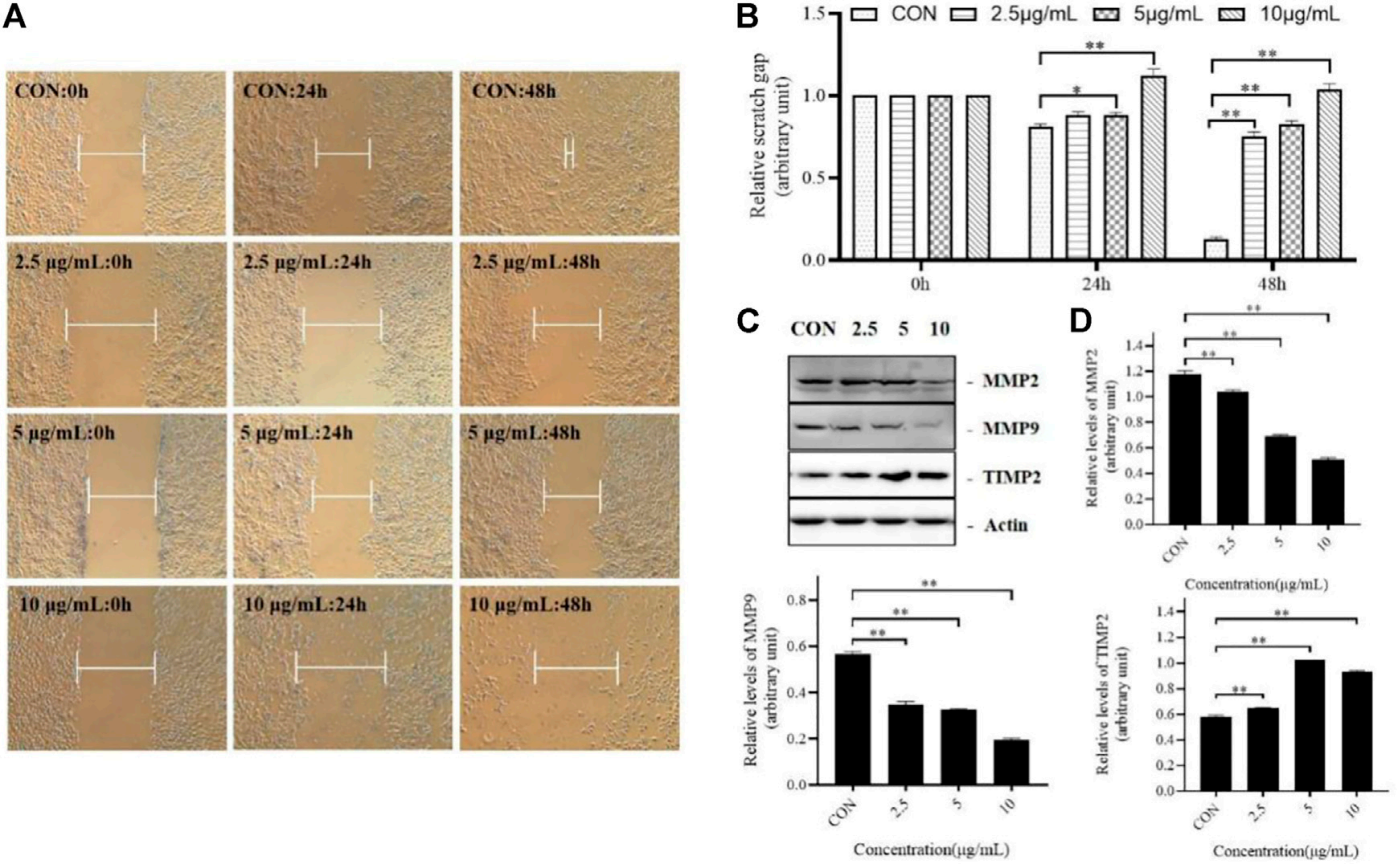
TTH Inhibits migration of H1975 Cells. **(A)** Scratch wound assay was conducted in H1975 cell. The distance indicated by while blunt-end line between unreached migrating cells was recorded at 0, 24, and 48 h post scratching; **(B)** Relative scratch gap was calculated as the ratio of the remaining scratch gap at given time point and the original gap at 0 µh, *p < 0.05, **p < 0.01 vs. control group; **(C)** Western blot showed the protein level of MMP-2, MMP-9 and TIMP-2 following the TTH treatment for 48 h. Actin served as an equal loading control; **(D)** Histogram summarized the results of **(C)**.Error bars represent means ± SEM of *n* = 3, *p < 0.05, **p < 0.01 vs. control group.

Abnormal cell migration can lead to normal physiological changes and diseases, such as the migration of vascular endothelial cells to the tumor to form new blood vessels, promoting tumor cell attachment and proliferation. Therefore, we investigated whether TTH could inhibit tumor cell migration. As shown in Figures 3A,B, after treatment 24 and 48 h, the scratch gap was significantly larger than that of the control group.

In this study, the protein expression levels of MMP-2, MMP-9 and TIMP-2 were detected. As shown in the Figures 3C,D, Group 1 was the control group, Group 2, Group 3 and Group 4 were treated with different concentrations of TTH. The results showed that the levels of MMP-2 and MMP-9 were decreased after TTH treatment, while the level of TIMP-2 was increased. These results suggested that the inhibition effects of TTH on cell migration were related to the downregulation of MMP-2 and MMP-9 and the upregulation of TIMP-2.

### Effect of TTH on the NF-κB and STAT3 pathway of H1975 cells

Recently, roles of NF-κB and STAT3 in many cancers have been extensively studied. They are two main factors for tumor monitoring and regulation of tumor angiogenesis and invasiveness in pre-tumor and malignant cells against apoptosis. The activation and interaction of these two pathways play an important role in communication between cancer cells and inflammatory cells. We investigated the effects of TTH on NF-κB and STAT3 signaling pathways, and detected the expression of NF-κB, p-STAT3, and STAT3.

**FIGURE 4:**
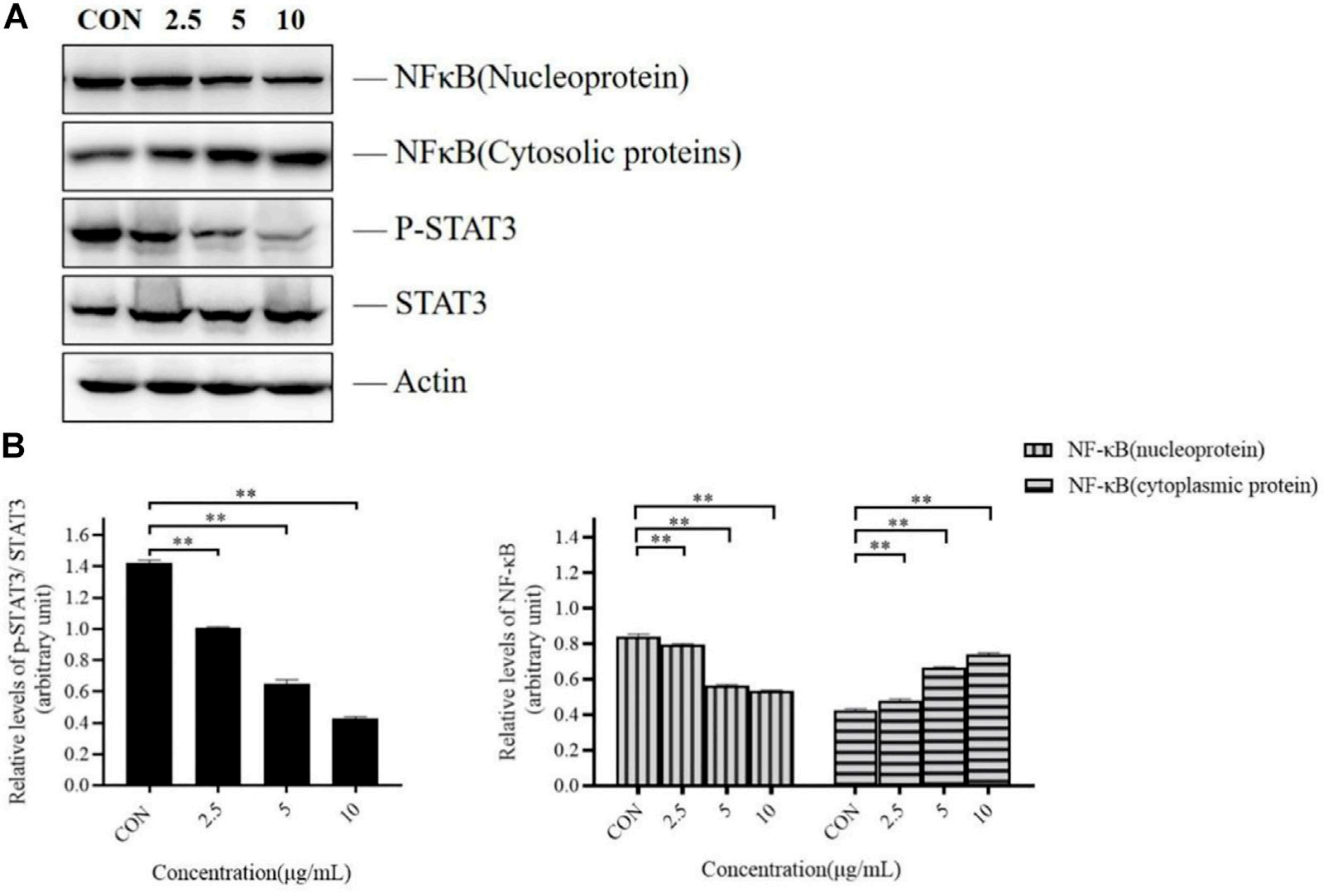
Effects of TTH on the NF-κB and STAT3 pathway of H1975 cells. **(A)** Cell lysates were analyzed by immunoblotting and quantified. Representative immunoblots of STAT3 and NF-κB were shown, with actin as the loading control. **(B)** Histogram summarized the results of **(A)**. Error bars represented means ± SD of *n* = 4. *p < 0.05 and **p < 0.01 vs. control group.

As presented in Figure 4, TTH treatment significantly reduced phosphorylation level of STAT3 in H1975 cells, but the total STAT3 protein level had no significant change. However, the expression of NF-κB (nucleus) was decreased and the expression of NF-κB (cytoplasm) protein was increased, when compared with untreated control cells (*p* < 0.05). These results suggested that the inhibition effects of TTH on H1975 may be related to NF- κB and STAT3 signal pathway.

## Discussions

As a traditional Chinese medicine, *H. diffusa* was used in many classic prescriptions for cancer treatment. However, the chemical compositions of traditional Chinese medicine are complex and their targets are diverse. Therefore, we investigated the effects of its active ingredient TTH and possible mechanisms on NSCLC. Tumor cells can proliferate indefinitely and destroy normal cell tissue. Oleanolic (OA) and ursolic acids (UA), which are TTH constituents, possess weak anti-inflammatory and anti-tumor properties. Previous studies have demonstrated that the IC_50_ of OA and UA is found for H1975 at 16 and 14 µg/ml. By using MTT and flow cytometry analysis, IC50 value of TTH was 10 µg/ml against H1975 (Figure 1A) and increase the number of cells in G0/G1 (Figures 1B,C). Ki67 is a proliferating cell associated antigen. Its function is closely related to mitosis and is indispensable in cell proliferation. Immunofluorescence results showed that TTH downregulated the expression of KI-67 and inhibited cell proliferation (Figure 1D). These results shown that H1975 cells were inhibited more effectively by TTH than OA or UA. The apoptotic cells that undergone both early and late apoptosis were determined by flow cytometry using Annexin V-FITC and PI staining, respectively. The results indicated that TTH could induce H1975 apoptosis (Figures 2A,B), but it didn’t increase compared with UA alone (Wang et al., 2020).

Caspase3 is a protease that plays a central role in the executive phase of apoptosis only when it is activated. Cleaved caspase3 is the active form of caspase3. It hydrolyzes on the conserved aspartic acid residues to produce two large and small subunits, which dimerize to form an active enzyme. Bax is necessary for the permeability of mitochondrial outer membrane and can be inhibited by the anti-apoptotic protein Bcl-2. TTH can reduce the expression level of apoptosis-related protein Bcl-2 and upregulate the expression level of pro-apoptotic protein Bax, but it does not have much effects on the activation of Casepase3 (Figures 2C,D).

Cell migration is a key link in pathological development such as inflammation and tumor. Results of cell scratch experiments showed that TTH inhibited cell migration (Figures 3A,B). Yang et al. (2019) also found that UA could inhibit migration of H1975, but the effect was not as optimistic as TTH treatment. There are many kinds of proteases that affect cell migration. Matrix metalloproteinases are the most closely related proteinases in tumor metastasis, which can degrade extracellular matrix (ECM), promote cell proliferation, growth and migration, resulting in uncontrollable cell feedback disorders, and promote the formation of new blood vessels and malignant development. MMP-2 and MMP-9 are the most direct and important proteases in tumor metastasis, and TIMP-2 is a tissue inhibitor of MMP-2. Other studies have shown that UA can inhibit cell migration by regulating MMP2 and MMP9 (Ruan et al., 2019), our results are consistent with UA. Results of TTH showed that the expression of migration-related proteins MMP-2 and MMP-9 were decreased, and upregulated the expression of TIMP-2 (Figures 3C,D), Taken together, these results suggested that the possible inhibitory effects of TTH on H1975 were depended on inhibiting cell migration, proliferation and promoting cell apoptosis. Thus, the above results suggest that TTH has better ability to inhibit both proliferation and migration of tumor cells than triterpene treatment alone.

NF-κB and STAT3 pathways play important roles in various tumorigenesis. In order to explore their roles with NSCLC, we did the following experiments. It has been studied in the literature that STAT3 is activated in EGFR mutant NSCLC (Chaib et al., 2017), Western blot analysis showed that the phosphorylation level of STAT3 decreased (Figures 3A,B), Orozco-Morales M (Orozco-Morales et al., 2021) found that S-allylcysteine (SAC) can reduce NF-κB expression in H1975 cells, thereby inhibiting tumor cell proliferation and inducing apoptosis. Previous studies (Xu et al., 2019) have demonstrated that the level of NF-κB (nucleus) reduced on H1975, and the level of NF-κB (cytoplasm) protein increased after Overexpression of TRIM13,which was consistent with the result of TTH(Figures 3B,C). These results indicated that the inhibitory effects of TTH on H1975 can be related to these two pathways. Although this study only reveals the tip of the iceberg, people firmly believe that *H. diffusa* has its anti-cancer activity through a variety of mechanisms. *H. diffusa* has broad application prospects due to its chemical components and pharmacological effects against malignant tumors. We should further study other chemical components of *H. diffusa* to provide useful information for finding more effective anti-cancer drugs.

## Conclusion

The mechanisms of related pathways in cancer cells are complex, and sometimes it is necessary to regulate one or more related pathways to achieve the effects of anti-cancer. To sum up, our study showed that TTH has a significant inhibitory effect on H1975 by acting on the NF-κB or STAT3 signal pathway. These findings are helpful for the treatment of NSCLC in the future. The anti-tumor effects of *H. diffusa* are still in the stage of experimental research, and the specific molecular mechanism of anti-tumor remain unclear. Therefore, our team will enhance research in this area to inform further studies and clinical applications of this drug.

## Data Availability

The original contributions presented in the study are included in the article/supplementary material, further inquiries can be directed to the corresponding author.
